# Intussusception after Laparoscopic Gastric Bypass Surgery: An Underrecognized Complication

**DOI:** 10.1155/2012/464853

**Published:** 2012-09-06

**Authors:** Smit Singla, Brandon A. Guenthart, Lauren May, John Gaughan, John E. Meilahn

**Affiliations:** Department of Surgery, Temple University Hospital, 3401 N. Broad Street, Philadelphia, PA 19140, USA

## Abstract

*Introduction*. Intussusception after bariatric surgery is an uncommon complication that is now being frequently reported. Most people consider dysmotility to be the causative mechanism in the absence of obvious etiology. *Material and Methods*. A worldwide search identified literature describing intussusception after bariatric surgery. We also included our own patients and analyzed information regarding demographic profile, risk factors, presentation, diagnosis, and post treatment course. *Results*. Seventy one patients were identified between 1991 and 2011. Majority of the affected patients were females (*n* = 70, 98.6%); median time to presentation after gastric bypass surgery was 36 months. Most patients presented with abdominal pain, nausea and vomiting, but without obvious peritonitis. Sixty eight patients (96%) required surgery; 48 (70.6%) underwent revision of anastomosis, 16 (23.5%) had reduction without resection, while 4 patients (5.9%) had plication only. Amongst these, most patients (*n* = 51, 75%) were found to have retrograde intussusception. Post-operatively, 9 patients presented with recurrence (range, 0.5–32 months). Five patients, who had earlier been treated without resection, eventually required revision of the anastomosis. There was no mortality noted. *Conclusion*. Intussusception after bariatric surgery is uncommon and its diagnosis is based on a combination of physicial, radiological and operative findings. An early surgical intervention reduces morbidity and prevents recurrence.

## 1. Introduction

Currently, it is believed that about one-third of the adult population in United States is obese, and this percentage is rising. As a result, we are witnessing a concurrent increase in the number of bariatric procedures performed for treating obesity in this country [1]. For many, weight loss surgery is the treatment modality of choice for the severely obese [2]. It has been shown that surgical interventions significantly improve the quality of life and reduce long-term morbidity and mortality [3]. The data collected over an 18-year period (1987–2004) from the International Bariatric Surgery Registry shows that more and more people are choosing surgery, and those undergoing surgery are now older and much heavier [4]. Although there are obvious benefits, surgery is certainly not without risks. As many as 25% of patients undergoing weight loss surgery require repeat surgery, either due to complications or failed weight loss. These patients are particularly at high risk, as the morbidity following these reoperative procedures is often high (9–22%), and mortality is not insignificant (0–1.4%) [5]. 

**Figure 1:**
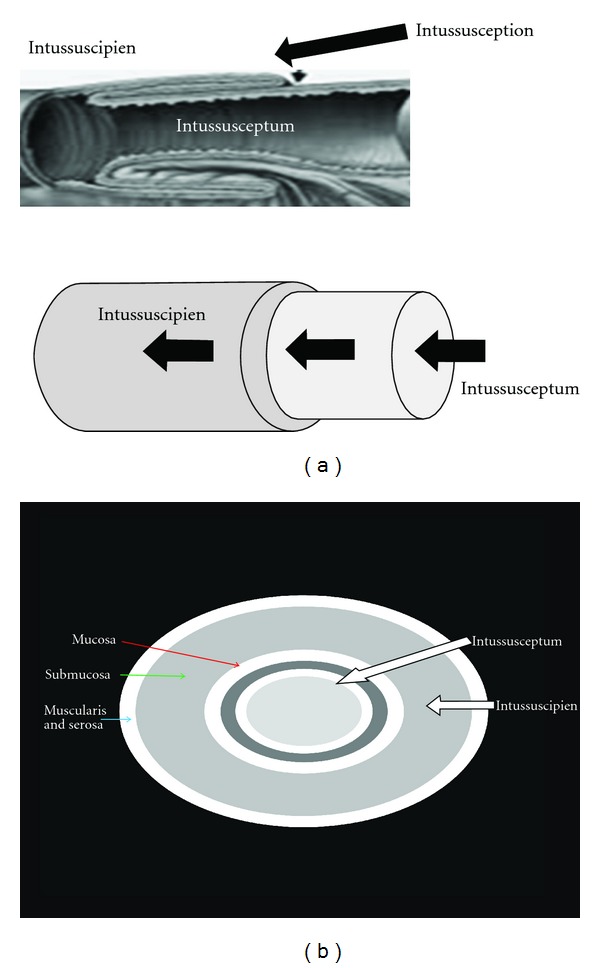
(a) Illustration of intussusception. (b) Target sign: it indicates hyperemia of mucosa, muscularis, and serosa with submucosal edema. The high attenuation of mucosa, muscularis, and serosa is due to contrast enhancement, while the low attenuation of submucosa is believed to result from edema.

**Figure 2:**
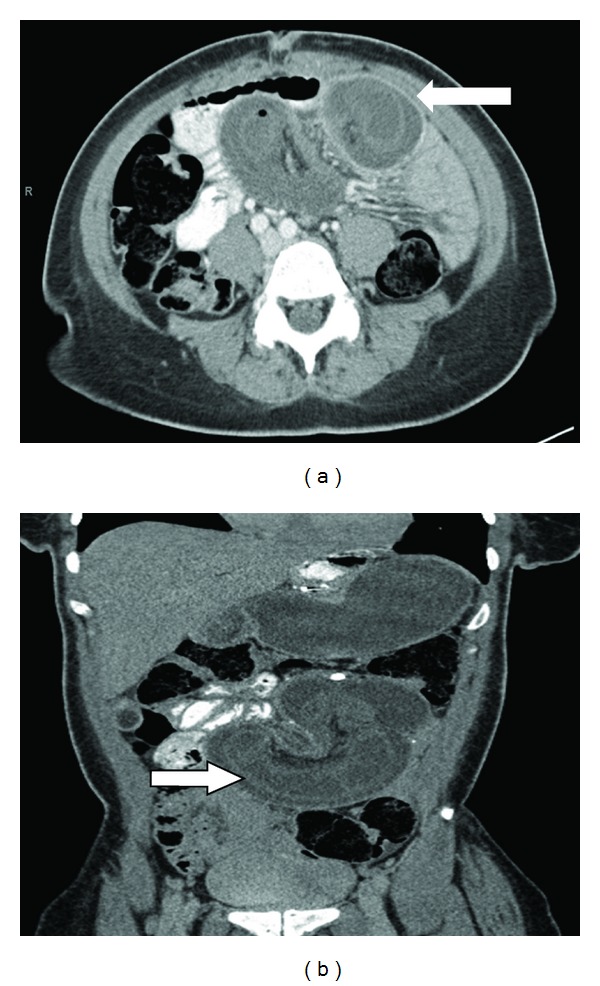
(a) Axial view of the CT scan showing intussusception with fat and blood vessels within the lumen of intestine (white arrow—target sign and pneumatosis). (b) Coronal view of the CT scan showing intussusception (white arrow—sausage-shaped thickened bowel wall).

**Figure 3:**
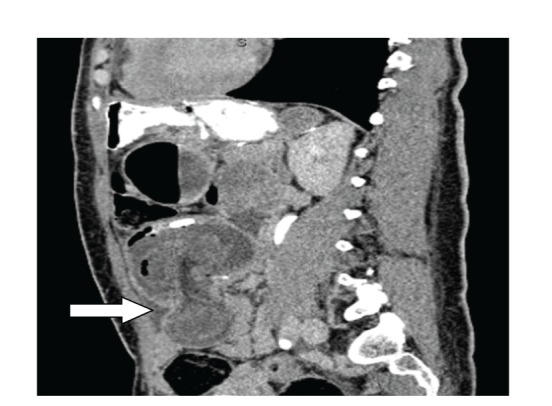
Sagittal view of the CT scan showing intussusception (white arrow—site of intussusception).

The reported incidence of intussusception following gastric bypass surgery is about 0.1–0.3% [6]. We believe that the true incidence is higher, and it will further rise in the next few years. This is because firstly, the number of gastric bypass surgeries performed is increasing rapidly, and secondly there is an increased awareness about this complication. More and more cases are being reported, and there are now better imaging modalities to detect this complication early. CT scans often reveal the classic “target sign” or “tube within a tube” sign (Figures 1(a) and 1(b)). Such is the sensitivity that many authors suggest that confirmatory radiological images should be obtained with CT scan prior to proceeding to the operating room [7, 8] (Figures 2(a), 2(b) and (3)). Still, other investigations such as plain film X-ray and ultrasound have been used to help make the diagnosis. The classic triad of abdominal pain, bloody stools, and a palpable mass is rarely seen in these cases of intussusception, and therefore, it is important to take a multimodality approach. The combined use of clinical history, physical exam, and radiographic images increases the sensitivity significantly and helps to plan the surgery in a more suitable time frame [8].

Although our ability to detect and treat intussusception following gastric bypass surgery has improved, its etiology remains somewhat unclear. Most people still believe that intussusception is related to dysmotility, which develops secondary to the development of ectopic pacemakers. Other proposed mechanisms include development of new lead points such as sutures or staple lines and focal nodal hyperplasia. However, in the vast majority of cases, no identifiable lead points or aberrations in anatomy are detected [7, 9, 10].

## 2. Material and Methods

A comprehensive search was conducted to identify the literature published worldwide including articles, reviews, case reports, and series and abstracts describing intussusception after gastric bypass surgery. We also included patients from our own clinical experience. We included all patients who underwent gastric bypass surgery for weight loss—both open and laparoscopic, confirmed diagnosis of intussusception—either preoperative or postoperative based on pathology. Patients with gastric bypass surgery for reasons other than weight loss, intussusception not associated with weight loss surgery, and diagnosis of intestinal obstruction due to causes other than intussusception were excluded in this review. 

The data was extracted using a structured form that included information regarding demographic profile, medical history, weight loss, clinical presentation, radiographic imaging, diagnosis, management, and posttreatment course in these patients (Table 1). 

## 3. Results

Seventy one patients were identified including seven patients from our own series, in 29 studies published worldwide between the years 1991 and 2011. The majority of patients identified were females (*n* = 70, 98.6%), with the median age of 35.5 years (range, 20–60 years). Sixty nine patients (97.2%) underwent Roux-en-Y gastric bypass, one patient received loop gastric bypass, and an other patient was treated with gastric bypass for weight loss, but the operative details were not available. Over the course of twenty years, seventy one patients were reported; however, the majority of these cases (*n* = 56, 79%) were reported after the year 2005.

The median time to presentation (from the time of weight loss surgery to development of intussusception) was 36 months (range, 6–133 months). Amongst the patients with data available, the mean excess weight loss was about 145 pounds. Most of the patients presented to the physician with complaints of diffuse abdominal pain, nausea, and vomiting. However, in nearly all patients, the abdomen was described as soft and without obvious peritonitis. A palpable mass was reported in 7 (9.8%) patients only. Amongst the 47 patients with detailed data available regarding imaging, CT scan was diagnostic in 38 (81%) patients. In other patients, the diagnosis was established based on findings from abdominal radiographs (*n* = 3), intraoperative (*n* = 3), small bowel follow-through (*n* = 2), and ultrasound (*n* = 1), respectively. 

**Figure 4:**
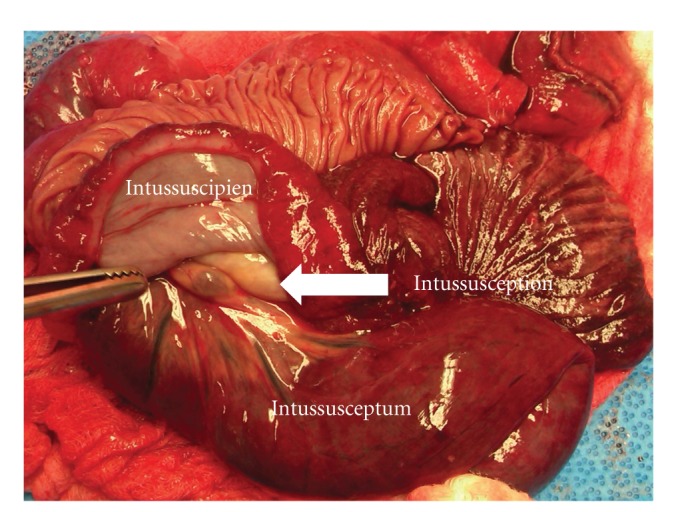
Resected specimen showing intussusception (note position of mesentery and blood vessels).

At the time of initial presentation, 68 (96%) patients underwent surgery, while 3 (4%) patients were treated nonoperatively. Amongst the patients treated operatively, 51 patients (75%) were found to have retrograde intussusception, 8 patients (11.8%) were reported to have antegrade intussusception, and the remaining 9 cases (13.2%) were not specified (Figure 4). Further, within this group, 48 (70.6%) patients underwent revision of anastomosis with small bowel resection, 16 (23.5%) patients had surgical reduction without resection, and the remaining 4 (5.9%) patients were treated with plication only. Amongst the three patients that were treated nonoperatively, one patient presented with repeated admissions, which eventually led to operative intervention, while the other two remained stable. Interestingly, both these patients who remained stable were diagnosed with intussusception based on findings obtained from abdominal radiographs. 

In the postoperative period, 20 patients developed complications ranging from pain and ileus to obstruction and recurrence (Table 2). Amongst these, nine (45%) patients were readmitted with recurrence (range, 0.5–32 months). Five of these patients with recurrence had been treated conservatively without bowel resection or reconstruction of anastomosis at the time of initial presentation/surgery. All these five patients were subsequently managed with surgical reexploration, small bowel resection, and reconstruction of the anastomosis. There were no further complications on followup. In spite of significant morbidity including multiple surgical interventions, there was no associated mortality reported. Given the small number of patients in this paper, a detailed statistical analysis has been withheld to prevent invalidation and bias.

## 4. Discussion

Intussusception in adults is relatively rare however; in patients undergoing gastric bypass surgery, the incidence is believed to be rising. Our analyses pose several questions that need to be answered: what are the risk factors? What is the etiology and why are females more commonly affected as compared to males? And what is the appropriate management of patients presenting with intussusception after gastric bypass surgery? To answer these questions, we looked at the problem in detail.

### 4.1. Risk Factors

The overall rate of complications associated with gastric bypass surgery is between 15% and 20% [11–13]. The spectrum of these complications is diverse, ranging from minor wound infection, nausea, and vomiting to anastomotic leak, pulmonary embolism, and death [11]. According to the available literature, surgeon experience, operative approach, body mass index (BMI), old age, and underlying medical conditions such as diabetes, hypertension, and sleep apnea are the major risk factors [11, 12, 14–16]. There is no specific gender or age predisposition, although in some studies, men and older patients were found to be more prone to complications [12, 17]. In our analysis, however, we found that nearly all patients affected with intussusception were females (*n* = 70, 98.6%). This percentage of affected females seemed to be significantly high. If we consider the fact that females are more likely to undergo gastric bypass surgery (4 out of 5 patients are females) [17, 18], and are also more likely to develop nonsincegastric bypass associated primary pathologic intussusception (55% in females and 45% in males) [19], the percentage of females developing intussusception after surgery may still exceed the likelihood that this was due to chance alone. However, at this stage given the small number of patients in our analysis, this may be considered an observation rather than a fact.

The majority of patients identified in our analysis were young with a median age of about 35.5 years. However, since most of the patients developing pathological primary intussusception or complications after gastric bypass surgery are relatively old [12, 17, 19], this group of patients are certainly in contrast to the conventional older patient population developing complications after gastric bypass surgery. Therefore, this raises a question whether younger patient population is at risk at developing this specific complication. Also, it was noted that most patients (97%) underwent Roux-en-Y gastric bypass surgery and had significant excess weight loss (150 pounds). Since Roux-en-Y gastric bypass causes significant weight loss and this weight loss has been found to be associated with significant thinning of the mesentery, it is believed by some that thinned mesentery offers less resistance to invagination once the intussusception is initiated [7]. It can, therefore, be argued that a relative young age and a significant excess weight loss are contributing factors to the development of intussusception after weight loss surgery. 

In summary, female gender, a relative young age, and significant excess weight loss after gastric bypass surgery may be considered as potential risk factors for the development of intussusception after gastric bypass surgery.

### 4.2. Etiology

The etiology for developing intussusception after gastric bypass appears more complex than previously thought. To date, the most widely accepted view has been that the creation of Roux limb disrupts the natural intestinal pacemakers in the duodenum and allows for the formation of ectopic pacemakers or migratory motor complexes in the Roux limb. It is believed that the electric potential generated by these ectopic pacemakers migrates in both the distal as well as the proximal limbs. This creates an area or segment of dysmotility, which according to some authors is responsible for developing intussusception in these patients [7, 10]. Researchers have also attributed the phenomenon of “Roux stasis syndrome” and the resultant delayed emptying to this alteration in motility [10]. Animal studies replicating Roux-en-Y gastric bypass construction have shown that suppression of these ectopic pacemakers by either electrical pacing or by using an “uncut roux” prevents stasis by maintaining enteric myoneural continuity [20]. 

It is our belief that the etiology of intussusception after gastric bypass is multifactorial and occurs due to the combination of the following: (1) disruption of the natural pacemakers. In the process of creating the Roux limb, the distal jejunum is separated from the proximal jejunal pacemaker during transection. This leads to a decreased pacesetter potential in the distal Roux limb and causes activation of the ectopic pacemakers in this limb. These ectopic pacemakers generate new pace-setting potentials that travel in both distal as well as proximal direction, resulting in delayed emptying and stasis of the Roux limb; (2) thinning of the mesentery. Substantial weight loss causes potential thinning of the mesentery around the intestine. This leads to a decreased cushion effect and increased bowel mobility around the roux limb and the jejunojejunostomy site, thereby creating a zone of instability. 

The combination of these two factors is believed to increase the risk of telescoping and intussusception and accentuate abnormal waves of dysmotility. This may explain why there is a delay in presentation and why most patients with this condition have lost a substantial amount of weight. Still, more analyses need to be made between patients with substantial weight loss from gastric bypass (Roux-en-Y) and others to determine if rates of intussusception show a statistically significant difference. 

### 4.3. Clinical Management

The majority of patients presented with nonspecific abdominal symptoms including diffuse abdominal pain, nausea, and vomiting. Interestingly, in nearly all of these patients, the abdomen was found to be soft, nonrigid, and without obvious peritonitis or any palpable mass (seen only in 7 patients). Further, we observed that in our series, most of the patients had nonspecific laboratory findings/values, without any indication or reflection on the underlying pathology in these patients. Since both physical examination and initial laboratory investigations were nonspecific and did not relay the appropriate information on the severity of the underlying pathology to the clinicians, we argued that the onus of diagnosing intussusception was dependent on further radiological investigations. 

We found that CT scan was the diagnostic study of choice in majority of patients studied. Most patients were found to have been investigated with more than one radiological investigation; however, the diagnosis was not established until the CT scan was completed. It may therefore be prudent to argue here that the CT scan is not only sensitive, but is also reliable in establishing the diagnosis early, and thus, in potential high-risk patients (females, young age, and significant excess weight loss), CT scan should take precedence over other investigations in diagnosing intussusception.

As regards the treatment, it is clear that surgical intervention is warranted early. However, in deciding how to operate, there is room for discussion. Some authors have suggested that simple reduction without resection is safe, while others have opted to proceed with resection of the bowel to prevent reoccurrence. Obviously, in cases that necessitate resection (bowel ischemia or necrosis), the latter is the treatment of choice. We found in our analysis that the majority of patients required small bowel resection and revision of the anastomosis. Those patients who were initially not treated with resection/revision subsequently developed recurrence and had to be operated again. 

Within our clinical experience, we found that the operative technique (open or laparoscopic), length of the limb, or the type of suture material/staplers made no difference in outcome. As long as the patients were treated with resection/revision, they did not develop recurrence. With regards how the revision is done, it is a matter of debate until more information becomes available. We treated our patients both laparoscopically and with open technique. However, because of the limited number of small patients and lack of statistical validation, these findings must be considered in light of clinical experience at this stage.

## 5. Conclusion

The diagnosis of intussusception in adults is relatively rare; however, we are noticing an increase in the incidence of this complication in patients who have undergone gastric bypass surgery. At present, the etiology is not very well understood, and most believe that dysmotility due to the development of ectopic pacemaker plays a crucial role in creating an unstable zone that predisposes to telescoping of the bowel. Further, the thinning of mesentery due to excessive weight loss decreases the “cushion effect” and potentially augments the unstable zone. Female gender, relative young age, and loss of significant amount of excess weight loss are potential risk factors for developing intussusception. 

The diagnosis is often difficult and not straightforward. This is because the initial physical examination and laboratory investigations are nonspecific. Further, it has been noted that plain X-rays and ultrasound are generally nonconfirmatory and can potentially blur the clinical picture further. Therefore, we propose a low threshhold for multimodality approach using a combination of initial examination, CT scan, and early surgical intervention to aid in diagnosis as well as provide optimal treatment. 

We believe that surgical intervention should entail bowel resection and revision of anastomosis as it prevents recurrence. As regards the technique is concerned, we will leave it at the discretion of the individual surgeon.

## Figures and Tables

**Table 1:** Summary of patient profile.

Patient number	Year of publication	Age	Gender	Initial surgery	Time to presentation (in years)	Diagnosis	Type of intuss.	Operation	Death	Post-op readmit
1	1991	31	F	Roux-en-Y	7	US	RINT	SBR	No	Yes
2	1996	40	F	Roux-en-Y	5	CT scan	INT	SBR	No	No
3	1996	35	F	Roux-en-Y	3	CT scan	INT	SBR	No	No
4	1996	36	F	Roux-en-Y	4	UGI	INT	SBR	No	No
5	2000	40	F	Roux-en-Y	5	CT scan	INT	SBR	No	No
6	2000	27	F	Roux-en-Y	4	X-ray	INT	SBR	No	No
7	2004	30	F	Roux-en-Y	3	CT scan	RINT	SBR	No	No
8	2004	30	F	Roux-en-Y	2	CT scan	RINT	SBR	No	No
9	2004	44	F	Roux-en-Y	1	CT scan	AINT	SBR	No	No
10	2004	33	F	Roux-en-Y	1.5	CT scan	AINT	SBR	No	No
11	2004	47	F	Roux-en-Y	2	CT scan	RINT	SBR	No	No
12	2004	36	F	Roux-en-Y	5	UGI	RINT	SBR	No	No
13	2004	48	F	Roux-en-Y	2	UGI	RINT	SBR	No	No
14	2004	39	F	Roux-en-Y	2	—	RINT	SBR	No	No
15	2004	49	F	Roux-en-Y	2.5	CT scan	RINT	SBR	No	No
16	2006	48	F	Roux-en-Y	1.5	CT scan	RINT	SBR	No	No
17	2006	33	F	Roux-en-Y	4	CT scan	RINT	SBR	No	No
18	2006	37	F	Roux-en-Y	3	CT scan	INT	SBR	No	No
19	2007	31	F	Roux-en-Y	1	Intra-op	AINT	Reduction	No	No
20	2007	44	F	Roux-en-Y	2.5	Intra-op	AINT	Reduction	No	No
21	2007	27	F	Roux-en-Y	3.5	Intra-op	AINT	Reduction	No	No
22	2007	35	F	Roux-en-Y	1	X-ray	RINT	SBR	No	No
23	2007	35	F	Roux-en-Y	4	CT scan	RINT	Reduction	No	No
24	2007	27	F	Roux-en-Y	3	X-ray	AINT	Reduction	No	Yes
25	2007	28	F	Roux-en-Y	1.5	CT scan	RINT	SBR	No	No
26	2007	58	F	Roux-en-Y	3	CT scan	INT	Reduction	No	No
27	2007	44	F	Roux-en-Y	6	CT scan	INT	SBR	No	No
28	2007	31	F	Roux-en-Y	3	CT scan	RINT	SBR	No	No
29	2008	46	F	Roux-en-Y	5	CT scan	RINT	SBR	No	No
30	2008	39	F	Roux-en-Y	4	CT scan	RINT	SBR	No	No
31	2008	51	F	Roux-en-Y	2	CT scan	RINT	SBR	No	No
32	2008	20	F	Roux-en-Y	1.58	—	RINT	SBR	No	No
33	2008	20	F	Roux-en-Y	1.83	—	RINT	SBR	No	No
34	2008	25	F	Roux-en-Y	5	—	RINT	SBR	No	No
35	2008	36	F	Roux-en-Y	5.17	—	RINT	SBR	No	No
36	2008	29	F	Roux-en-Y	3.25	—	RINT	SBR	No	No
37	2008	41	F	Roux-en-Y	4.25	—	RINT	SBR	No	No
38	2008	38	F	Roux-en-Y	1.5	—	RINT	SBR	No	No
39	2008	36	F	Roux-en-Y	3.83	—	RINT	SBR	No	No
40	2008	32	F	Roux-en-Y	4.17	—	RINT	Reduction	No	No
41	2008	29	F	Roux-en-Y	1.33	—	RINT	SBR	No	No
42	2008	20	F	Roux-en-Y	2.33	—	RINT	SBR	No	No
43	2008	25	F	Roux-en-Y	1.58	—	RINT	SBR	No	Yes
44	2008	33	F	Roux-en-Y	10	—	RINT	Reduction	No	Yes
45	2008	28	F	Roux-en-Y	11.08	—	RINT	Reduction	No	Yes
46	2008	50	F	Other	5	—	RINT	Plication	No	No
47	2008	36	F	Roux-en-Y	0.67	—	RINT	Plication	No	No
48	2008	41	F	Roux-en-Y	5.83	—	RINT	Plication	No	Yes
49	2008	25	F	Roux-en-Y	9	—	RINT	Plication	No	Yes
50	2008	34	F	Roux-en-Y	9.17	—	RINT	SBR	No	No
51	2008	50	F	Roux-en-Y	0.5	—	RINT	SBR	No	No
52	2008	23	F	Roux-en-Y	3.67	—	RINT	SBR	No	No
53	2008	25	F	Roux-en-Y	2.33	—	RINT	SBR	No	No
54	2008	32	F	Roux-en-Y	2.33	—	RINT	SBR	No	Yes
55	2009	60	F	Roux-en-Y	4	CT scan	RINT	SBR	No	No
56	2009	25	F	Roux-en-Y	5	CT scan	RINT	Reduction	No	No
57	2009	32	F	Roux-en-Y	3	CT scan	RINT	Reduction	No	No
58	2009	27	F	Roux-en-Y	1.5	CT scan	AINT	Reduction	No	No
59	2009	33	F	Roux-en-Y	1	CT scan	RINT	SBR	No	No
60	2009	51	F	Roux-en-Y	2	CT scan	RINT	SBR	No	No
61	2009	37	F	Roux-en-Y	5	CT scan	RINT	SBR	No	No
62	2010	27	F	Roux-en-Y	2	CT scan	AINT	Reduction	No	No
63	2010	42	F	Roux-en-Y	0.75	CT scan	RINT	Reduction	No	No
64	2010	25	F	Roux-en-Y		CT scan	RINT	SBR	No	No
65	2011	36	F	Roux-en-Y	3	CT scan	—	Non-op	No	No
66	2011	28	M	Roux-en-Y	8	CT scan	—	Non-op	No	Yes
67	2011	29	F	Roux-en-Y	6	CT scan	RINT	Reduction	No	Yes
68	2011	31	F	Roux-en-Y	8	CT scan	—	Non-op	No	No
69	2011	44	F	Roux-en-Y	1	CT scan	RINT	Reduction	No	Yes
70	2011	47	F	Loop GBP	11	CT scan	INT	Rev. loop	No	Yes
71	2011	41	F	Roux-en-Y	5	CT scan	RINT	SBR	No	Yes

**Table 2:** List of complications after initial treatment for intussusception.

Complication	Number of patients
Recurrence with intussusception	9
Pain	4
Ileus	3
Bleeding	1
Marginal ulcer	1
Obstruction due adhesions	1
Intra-abdominal abscess	1
